# Using the Health Belief Model to Explore Behavior Change in the Community to Improve Blood Pressure Control: A Qualitative Study

**DOI:** 10.1177/01939459261432411

**Published:** 2026-04-24

**Authors:** Pataporn Bawornthip, Jo Mcdonall, Decha Tamdee, Andrea Driscoll, Anastasia Hutchinson

**Affiliations:** 1 Faculty of Health, School of Nursing and Midwifery, Deakin University, Geelong, VIC, Australia; 2 Centre for Quality and Patient Safety Research, Deakin University, Geelong, VIC, Australia; 3 Faculty of Nursing, Chiang Mai University, Chiang Mai, Thailand; 4 School of Nursing and Midwifery, La Trobe University, Melbourne, VIC, Australia; 5 Centre for Quality and Patient Safety Research-Monash Health Partnership, Deakin University, Geelong, VIC, Australia; 6 Faculty of Health-Institute for Health Transformation, Centre for Quality and Patient Safety Research-Epworth HealthCare Partnership, Deakin University, Geelong, VIC, Australia

**Keywords:** Health Belief Model, hypertension prevention, hypertension, prehypertension, rural community, qualitative

## Abstract

**Background:**

Effective hypertension prevention requires understanding individual and community perceptions and responses to health promotion messages to inform culturally tailored interventions. This study aimed to explore the perceptions of local key stakeholders and consumers of the barriers and facilitators influencing the uptake of behavior change strategies that promote optimal blood pressure control.

**Methods:**

The Health Belief Model underpinned the design of this qualitative study. Three focus groups (N = 27), including community leaders (n = 9), health care workers (n = 10), and consumers (individuals with prehypertension and hypertension, family members; n = 8) were conducted. Purposive sampling based on recommendations from an advisory group was used to recruit participants. Data were analyzed using reflexive thematic analysis.

**Results:**

Four themes and 11 subthemes were generated including the following: (1) community perceptions of the rising prevalence of hypertension and its associated health and social consequences (perceived severity); (2) hypertension is asymptomatic, and as a consequence, there is little motivation for behavior change (perceived susceptibility); (3) contextual and cultural barriers challenging the adoption a healthy lifestyle in everyday life (perceived barriers); and (4) enhancing enablers and overcoming barriers to behavior change (perceived benefits, cues to action, and self-efficacy).

**Conclusion:**

Local stakeholders and consumers identified contextual and cultural barriers as the main obstacles to behavior change, highlighting the need for both community-level strategies to promote participatory public health policy and healthy environments and individual-level strategies to motivate behavior change and help individuals overcome barriers, thereby encouraging sustained change.

**Reporting Method:**

The Consolidated Criteria for Reporting Qualitative Research was used to instruct this report.

**Patient or Public Contribution:**

Patients and community stakeholders actively participated in the data collection process through in-person focus group discussions.

## Introduction

Hypertension is a leading cause of premature death worldwide and is associated with cardiovascular and cerebrovascular disease.^1^ The prevalence of hypertension is higher in low- and middle-income countries (LMICs), at 31.5%, than in high-income countries, 28.5%.^2,3^ In Southeast Asia, the prevalence of hypertension and prehypertension is increasing, especially in rural and remote communities^4-7^; for example, the prevalence of prehypertension in Thai people living in remote areas is estimated to be as high as 67.8%.^8^ Rural and remote communities are confronted with numerous barriers to accessing health information due to the limited number of health care providers, poor Internet connections, and the financial burden associated with travel to larger centers for care provision.^9,10^ Nurses and primary health care workers play a vital role in these communities through promotion of healthy lifestyle behaviors and implementation of preventive health initiatives.^11,12^

Health care workers face challenges in promoting preventive measures for hypertension due to the unique characteristics of Asian populations, such as the adoption of Western lifestyles, modernization, excessive salt intake, and increased salt sensitivity.^13^ In Southeast Asia, contextual, cultural, and socioeconomic factors—such as high-sodium traditional foods, food costs, limited healthy food options, adherence to family diets, and a lack of safe spaces for exercise—act as barriers to adopt of healthy lifestyle behaviors.^14-18^ Moreover, tropical climates and air pollution are also associated with reduced opportunities for physical activity.^18,19^ To address the burden of hypertension and reduce the prevalence of prehypertension in regions with unique characteristics, it is essential to explore individuals’ perceptions to design health promotion interventions that meet their specific needs.

The Health Belief Model (HBM) provides a useful and widely applied framework for explaining preventive health behaviors based on an individual’s perceived threat and the expected net benefit of recommended actions.^20-22^ According to the HBM, individuals are more likely to take action to prevent disease if they perceive themselves as susceptible and believe the condition has serious consequences and that adopting healthy behaviors could reduce their risk. In addition, individuals need to have self-efficacy to make lifestyle changes, the skills and resources to overcome barriers to change, and community supports to assist them in making changes.^23^

The HBM has been used to explain the relationship between the uptake of preventive behaviors and noncommunicable diseases (NCDs), including hypertension in Southeast Asia.^14,23-26^ A study conducted in Indonesia found that higher self-efficacy, perceived threats associated with uncontrolled hypertension, and perceived benefits had a positive effect on the uptake of preventive behaviors, while perceived barriers to behavior change had a negative impact.^26^ Similarly, a study in Malaysia showed that perceived barriers significantly decreased the uptake of preventive self-care behaviors among individuals with prehypertension.^14^ In Thailand, a systematic review illustrated that self-efficacy, perceived benefits, and barriers to lifestyle change, as well as communication with health care providers, influenced the uptake of preventive behaviors among adults with hypertension.^25^

Promoting behavior change to improve blood pressure control requires a deep understanding of both individual and community perceptions, along with their responses to health prevention messages. These insights are essential for codesigning and developing tailored, culturally sensitive interventions that support optimal blood pressure control. This qualitative descriptive study was conducted in rural northern Thailand and explored key stakeholders’ and consumers’ perceptions of the barriers and facilitators to adopting recommended lifestyle changes to decrease the burden of hypertension and its consequences in their community.

## Study Aim

We sought to explore the perceptions of local key stakeholders and consumers of the barriers and facilitators influencing the uptake of behavior change strategies to promote optimal blood-pressure control.

## Methods

### Design

This study used a qualitative exploratory descriptive study design. Focus groups with key community stakeholders were conducted in September 2024. The Consolidated Criteria for Reporting Qualitative Research checklist was used to structure this report.^27^

### Framework

The HBM suggests that people are more likely to take preventive action if they perceive a health threat as serious and themselves as susceptible, believe the action will be beneficial, and view the benefits as outweighing the barriers.^21^ The HBM consists of 4 perceptions (perceived susceptibility, perceived severity, perceived benefit, and perceived barriers), and 2 modifying factors, cues to action and self-efficacy.^28^

### Setting and Recruitment

#### Setting

The study was conducted in a Northern rural community located in Ta-Kwang subdistrict, Saraphi District, Chiang Mai Province, Thailand. Bankwae Health Promoting Hospital, a primary health care center in the Ta-Kwang subdistrict, provides holistic care to approximately 2700 residents across 7 villages, recognizing individuals as whole persons and the interdependence of biological, psychological, social, and spiritual dimensions of health.^29^ One focus group was conducted per day in a meeting room at Bankwae Health Promoting Hospital.

#### Recruitment

The total sample size for the focus groups was 27 (8-10 per group) and selected using purposive sampling. The project advisory group (head of health promoting hospital, health village volunteer, and municipal advisor) identified key stakeholders with experience in developing programs for the prevention and management of chronic disease in a community setting. The first focus group of community leaders consisted of 9 participants including 1 mayor of Ta-Kwang municipality, 1 deputy mayor of Ta-Kwang municipality, and 7 village leaders. For the second focus group of health care workers consisted of 10 participants including the head of the health promoting hospital, 1 nurse practitioner, a village health volunteer (VHV) leader, and 7 VHVs. The third focus group of consumers consisted of 8 participants including 2 people with prehypertension, 2 people with hypertension, and 4 caregivers or family members who care for people with prehypertension and hypertension. The researcher contacted potential participants via phone call and letter explaining the purpose of the study and inviting them to participate. Those who accepted the invitation and expressed willingness to participate were then formally recruited into the study. No one refused to participate in the study.

#### Definition of VHVs and Village Leaders

VHVs are trained individuals qualified to provide preventive, monitoring, and supportive care for people with chronic diseases in primary health care settings.^30^ Village leaders, or village headmen, are individuals who hold legal and public administrative responsibilities within local government areas, which are the smallest administrative units in Thailand.^31^

### Inclusion and Exclusion Criteria

Participants were eligible for inclusion if they were adults aged 20 years or older, were able to speak, communicate, read, and write in the Thai language, and were willing to provide written informed consent to participate in the study. Participants were required to have lived or worked in the Ta-Kwang subdistrict for at least 6 months. Eligible participants included community leaders, as well as individuals diagnosed with hypertension or prehypertension who had received treatment or health care services at Bankwae Health Promoting Hospital and their family members. Individuals with cognitive impairment that interfered with their ability to communicate effectively or to participate fully in the study were excluded.

#### Definition of Hypertension and Prehypertension

Prehypertension or hypertension was defined according to the Noncommunicable Disease Control Group Chiang Mai Provincial Public Health Office, Thailand (2023) as mean systolic blood pressure 130 to 139 and /or diastolic blood pressure 85 to 89 mmHg in prehypertension and mean systolic blood pressure 140 mmHg or/and diastolic blood pressure 90 mmHg in hypertension.

### Data Collection

The principal researcher (P.B.) conducted 3 focus groups using a discussion guide to lead conversations with local stakeholders. P.B. is a bilingual Thai female nurse practitioner who has completed a course in qualitative health research and has experience conducting research in rural communities. The research assistant who took field notes during the focus groups received 1 hour of training in qualitative research and note-taking. Before commencing the focus group discussion, P.B. explained the purpose and objectives of the research, and written informed consent was obtained from all participants. During the focus groups, P.B. used active listening and prompting techniques to encourage quieter participants to share their perspectives. Each focus group, conducted in Thai, lasted 60 to 90 minutes and was audio-recorded for later transcription. The transcripts were returned to participants for correction, but no further suggestions were made. The semi-structured interview guide includes 6 questions for community leaders, 9 questions for health care workers, and 14 questions for consumers (see Supplemental File, Table S1).

### Data Analysis

Reflexive thematic analysis is a widely used and theoretically flexible interpretive approach to qualitative data analysis that facilitates the identification, analysis, and interpretation of patterns or themes within a data set.^32^ Reflexive thematic analysis was conducted using the 6 steps.^33^

#### Step 1: Familiarization of Data Set

Author P.B. actively listened to the audio-recorded data and transcribed the recordings in Thai. The transcriptions were checked for accuracy by both P.B. and D.T. The Thai transcripts were then translated into English by P.B. for analysis. To ensure trustworthiness, both the Thai and English transcripts were reviewed by an independent bilingual Thai academic with expertise in both languages.

#### Step 2: Generation of Codes

Researchers generated codes (see Supplemental File, Figure S1) relevant to the research questions, capturing a range of meaning abstractions—from semantic (the manifest content of the data) to latent (the underlying meaning). Microsoft Excel was used to manage and organize the textual data.

#### Step 3: Generating Initial Themes

A deductive qualitative approach was used to guide the generation of themes. The codes were grouped to generate themes and subthemes based on the coded data.

#### Step 4: Developing and Reviewing Themes

P.B. reviewed and discussed the proposed themes, considering whether the themes provided a useful synthesis and aligned with the HBM, the categories represented themes or sub-themes, the quality of quotes supporting each theme, the boundaries of what each theme captured, and whether the data were too diverse or broad in range. A second member of the research team (D.T.), who is a native Thai speaker, cross-checked and verified the themes and subthemes.

#### Step 5: Refining, Defining, and Naming Themes

The researchers refined the specifics of each theme to ensure they accurately reflected the meaning of the data. Additionally, themes were named in a manner that was concise, informative, and memorable.

#### Step 6: Reporting of Findings

The researchers developed a report explaining the thematic structure and relationships between themes and subthemes, providing illustrative quotes to support the analysis.

## Results

Most participants were female (77.8%), with an average age of 60.6 years, and 48.5% had graduated from high school (Table 1). The community leader, health care worker, and VHVs had between 3 and 38 years of experience in their roles, with an average of 18.9 years. Consumer participants had been diagnosed with hypertension for 12 to 16 years (n = 2) or with prehypertension for 2 years (n = 2). In addition, 3 participants (family members) had experience caring for individuals with hypertension or prehypertension, with an average duration of 17 years.

**Table 1.** Characteristics of Participants (N = 27).

Characteristics	Data value
Sex, n (%)	
Female	21 (77.8)
Male	6 (22.2)
Age, y	
Mean (SD)	60.6 (9.5)
Range (Min-Max)	36-73
Education, n (%)	
Primary education	10 (37.0)
High school	13 (48.1)
Bachelor’s degree or over	4 (14.8)
Health care worker and community leader* (n = 20)	
Duration of position, y	
Mean (SD)	18.9 (14.1)
Range (Min-Max)	3-38
1-10, n (%)	8 (40.0)
11-20, n (%)	4 (20.0)
21 and over, n (%)	8 (40.0)
Consumer participants (n = 7)	
Duration of HT (years) (n = 2)	
Mean (SD)	14.0 (2.8)
Range (Min-Max)	12-16
Duration of PHT (years) (n = 2)	2
Family members** (n = 3)	
Duration of experience with hypertension or care individuals with HT or PHT	
Mean (SD)	17.00 (2.65)
Range (Min-Max)	15-20

Abbreviations: HT, hypertension; PHT, prehypertension; SD, standard deviation.

* Six health care workers and 9 community leaders had personal experience with HT, either themselves or through family members.

** Family members (N = 3), one of the family members is village health volunteer.

Four themes and 11 subthemes were generated from the data (Table 2) including the following: (1) Community perceptions of the rising prevalence of hypertension and its associated health and social consequences; (2) High blood pressure is asymptomatic, and as a consequence, there is little motivation for behavior change; (3) Contextual and cultural barriers challenging the adoption of healthy lifestyles in everyday life; and (4) Enhancing enablers and overcoming barriers to behavior change.

**Table 2.** Themes, Subthemes, and Representative of Quotes.

Health Belief Model component	Theme	Subthemes	Representative of quotes
Perceived severity	1. Community perceptions of the rising prevalence of hypertension and its associated health and social consequences	1.1. Hypertension is hidden but its consequences impact on family life and community	*Some individuals at risk have direct family members who also have hypertension. Hypertension hasn’t been the immediate cause of death, but many patients have experienced strokes. (FG1-CL-P7)* *It’s not decreasing (prevalence of hypertension and prehypertension); it’s either staying the same or increasing. It comes from eating habits. (FG2-HCW-P1)* *I’ve been to a black-and-white event (funeral). The village health volunteer then measured my blood pressure, and it was 200. After resting, it dropped to 195. The village health volunteer told me to go home and rest immediately. (FG3-C-P6)* *When I feel dizzy, I can’t participate in community events. I have to stay home. (FG3-C-P8)*
		1.2. The exponential growth in NCDs is made visible in our overcrowded clinics.	*There’s some overcrowding in the service area because the space for seeing the doctor is small, and people have to wait outside. After the examination with the senior doctor is finished, we have to dispense medicine, so there’s no time. (FG2-HCW-P1)* *Staffing is insufficient. . .sometimes the pharmacist arrives late. . .sometimes we have to provide services in the emergency room too, which makes us short-staffed to help with the NCD clinic. (FG2-HCW-P10)* *Sometimes I have an appointment, but I’m working. I ask my older sister to get my medicine for me because I don’t need to wait, which can take 3 to 4 h. (FG3-C-P2)*
Perceived susceptibility	2. High blood pressure is asymptomatic, as a consequence there is little motivation for behavior change	2.1. Community members do not perceive themselves to be at risk.	*“They focus on eating sweets. They enjoy it. They eat what they want. They don’t lose weight.” (FG2-HCW-P4)* *The blood pressure monitor said my readings were normal, so I didn’t think I was at risk. (FG3-C-P4)* *I can’t remember the lower one. I didn’t have any symptoms. (FG3-C-P7)*
		2.2. The importance of disease prevention is not widely understood, and people do not understand the consequences of NCDs.	*Some groups don’t care, saying that if they get sick, they’ll just die anyway. (FG1-CL-P7)* *They wait until their condition worsens before seeking medical attention, despite having health insurance and access to free annual check-ups at the hospital. (FG1-CL-P7)* *Patient is going to be. . .not aware. If they have the condition, there is medicine to take. (FG2-HCW-P9)*
Perceived barriers	3. Contextual and cultural barriers challenging the adoption of healthy lifestyles in everyday life.	3.1. Working patterns and busy lifestyle pose challenges to adopt healthy behaviors.	*“It might be due to working age, elderly age, working in factories. For eating, they have to buy for speed, it might come from the seasonings in the food”. (FG2-HCW-P1)* *“I don’t exercise as much because I’m busy with work. I mostly just walk.” (FG3-C-P8)*. *“Those who work in the industrial estate are unable to hear these announcements because they leave home before 6:30 AM.” (FG1-CL-P2)*
		3.2. Adverse weather conditions, disease outbreaks and lack of environmental safety make it difficult for individuals to maintain an exercise program.	*“They don’t have time to participate in activities. Additionally, they are unable to exercise due to the harvesting season and air pollution.” (FG1-CL-P10).* *Yes, there are exercise facilities. Usage tends to decrease during the rainy season. (FG1-CL-P3)* *There’s a fully equipped gym in my community, but the gym is empty and quiet. I feel a bit scared being alone there. (FG3-C-P2)* *We go cycling together, but now there are a lot of cars. So, we don’t go very often. (FG3-C-P6)* *Sometimes, when I bike, the dogs chase after me. In fact, some people have even fallen into the canal. (FG3-C-P8)*
		3.3. Traditional foods, particularly those served and seasoned during festivals, are delicious but often high in sodium and fat.	*“In our communities, we eat northern Thai food, which has seasonings like fermented fish (pla ra) and shrimp paste (ka pi).” (FG2-HCW-P1).* *The seasonings I use regularly are light soy sauce, oyster sauce, chicken broth, sweet soy sauce, fish sauce, fermented fish paste, and shrimp paste. People in our community eat like that, so it’s really difficult [to decrease sodium intake]. (FG3-C-P4)* “*I often go to community events and can’t always choose what to eat. There’s always high-fat curries and stir-fries menu.” (FG3-C-P6).* *“I heard at a health education training session on changing behaviours that there are low-sodium seasoning products, but they’re not sold in my area.” (FG3-C-P8)*
		3.4. Lack of trust in information and low adherence to advice from nurses and village health volunteers.	*If the doctor tells me that I am risk of hypertension, then I will stop eating sweet, fatty, salty, and spicy foods. If I am healthy, I would eat them all. (FG3-C-P4).* *The information that patients tell us [nurses] is different from what they tell the “mor yai” (senior doctor). Patients tend to trust the “mor yai” more than the “mor noi” (nurse), which makes it difficult to provide information. (FG2-HCW-P1)* *I told his wife to take him to the hospital. . .later his son came and said, “It’s just a normal part of aging” It was as if he was telling us it is not our business. However, 7 days later, he couldn’t get up. (FG1-CL-P7)*
Perceived benefits, cues to action and self-efficacy	4. Enhancing enablers and overcoming barriers to change behavior.	4.1. Benefits of complying with blood pressure control	*I received it [health information] when I came to pick up medication at the health-promoting hospital. (FG3-C-P8)* *I received information during my visits to the primary health care, sometimes at Hospital. There are hours where nurses provide education. (FG3-C-P6)* *I’ve been receiving health education training continuously because I’m a patient. (FG3-C-P3)*
		4.2. Tailored health education is essential to promote engagement in behavior change.	*“Actually, there should be [health counseling/education], but due to the situation at the work site, it’s not possible.” (FG2-HCW-P1).* *“I’ve never received training” (FG3-C-P4)* *“I’ve participated in many in programs, but I can’t remember all the details” (FG3-C-P8)*
		4.3. Individual ability to reduce barriers to exercise.	*I prefer low-intensity physical activity. I have a stationary bike at home, so I don’t need to use gym equipment. (FG3-C-P4)* *I used to exercise regularly, but now that I’m older, I’ve switched to walking. I used to walk 2 kilometers a day, but I stopped because my knees got worse. Now, I do arm circles for 15-20 min every morning. (FG3-C-P6)*

Abbreviations: FG1-CL, Focus Group 1 (community leaders); FG2-HCW, Focus Group 2 (health care workers); FG3-C, Focus Group 3 (consumers); NCDs, Noncommunicable disease; P, order of participants in each group.

### Theme 1: Community Perceptions of the Rising Prevalence of Hypertension and its Associated Health and Social Consequences

People in the community recognized that the number of individuals with hypertension was increasing and that the complications of uncontrolled blood pressure could be life-threatening. Moreover, the rising prevalence of hypertension impacts the delivery of health care services due to the increasing number of individuals with NCDs, especially hypertension.

#### Subtheme 1.1: Hypertension is Hidden but its Consequences Impact on Family Life and Community

The prevalence of hypertension and prehypertension is increasing and is influenced by both modifiable and non-modifiable factors. Factors such as a family history of hypertension were recognized as a contributing factor. For example, one community leader shared that his father had hypertension and was later diagnosed with a stroke. Health care workers noted that the number of individuals with hypertension and prehypertension is increasing and that many of them have unhealthy dietary behaviors.

Furthermore, Northern Thai rural communities have a unique tradition and culture that reflects generosity and unity among community members. Two consumers reported that they could not participate in a community event due to uncontrolled hypertension. One consumer was screened during the activity and found to have high blood pressure and, due to the elevated reading, was unable to participate. The other consumer was unable to participate because of dizziness.

#### Subtheme 1.2: The Exponential Growth in NCDs is Made Visible in our Overcrowded Clinics

Health care workers recognized that the number of people with hypertension and prehypertension was increasing, but the number of health care providers and the available space for support remained the same. This resulted in longer waiting times and overcrowding in community clinics. One consumer noted that they could not wait to collect their medication because they needed to go to work, so they asked a relative to attend instead.

### Theme 2: High Blood Pressure is Asymptomatic; Consequently, There is Little Motivation for Behavior Change

The uptake of prevention strategies is currently hindered by a lack of perceived personal risk and a general misconception regarding the importance and impact of NCD prevention.

#### Subtheme 2.1: Community Members do not Perceive Themselves to be at Risk

None of the consumer participants with prehypertension reported experiencing any signs or symptoms indicating high blood pressure. Two consumers with prehypertension reported that although they measured their blood pressure at home or in hospital, they did not understand the significance of the readings. This result highlights that if individuals perceive themselves as healthy, they are likely to continue engaging in the same behaviors. Health care workers reported that some individuals with prehypertension continue to consume high-sugar foods.

#### Subtheme 2.2: The Importance of Disease Prevention is not Widely Understood, and People do not Understand the Consequences of NCDs

Community leaders and health care workers noted that individuals with prehypertension demonstrated limited concern and awareness regarding health screening, despite identifiable risk factors and the availability of free services. Some actively avoided being diagnosed with hypertension. Among those who were diagnosed, there was a tendency to rely solely on medication without adopting recommended lifestyle modifications.

### Theme 3: Contextual and Cultural Barriers to Adoption of a Healthy Lifestyle in Everyday Life

When participants perceived obstacles to making recommended lifestyle changes, they were discouraged from taking action to decrease their health risks.

#### Subtheme 3.1: Working Patterns and Busy Lifestyles Pose Challenges to Adopting Healthy Behaviors

Health care workers recognized that the working population often led a fast-paced and demanding lifestyle, frequently relying on street food or ready-to-eat meals due to limited time for meal preparation. One consumer noted that physical inactivity was also prevalent, as many individuals reported feeling fatigued and exhausted after work. Additionally, early work hours reduced their exposure to health information and limited opportunities to engage with available health care services.

#### Subtheme 3.2: Adverse Weather Conditions, Disease Outbreaks, and Lack of Environmental Safety Make it Difficult for Individuals to Maintain an Exercise Program

Environmental factors also appeared as critical restrictions. Participants specifically highlighted that rain, flooding, high levels of air pollution (fine particulate matter [PM2.5]) during the rainy and summer seasons, and the COVID-19 outbreak limited people’s ability to engage in outdoor physical activity. Community leaders noted that this occurred despite the community’s preference for, and the availability of, exercise-supporting resources such as swimming pools and fitness equipment. Concerns about environmental safety played a crucial role in discouraging exercise participation. For example, some consumers expressed fear of using the community gym due to feelings of insecurity and outdoor exercise activities were considered difficult and unsafe, due to the traffic and the presence of stray dogs.

#### Subtheme 3.3: Traditional Foods, Particularly those Served and Seasoned During Festivals, are Delicious but often High in Sodium and Fat

Participants reported that traditional Thai local foods, commonly consumed in daily life and frequently served at community events, contain a variety of condiments that are typically high in fat, salt, and sugar. Moreover, consumers noted that when they participate in community activities such as weddings, funerals, and house-warming events, they often find it difficult to refuse such food offerings due to cultural norms and social expectations. One participant also commented that low-sodium products are not readily available for purchase within the community.

#### Subtheme 3.4: Lack of Trust in Information and Low Adherence to Advice from Nurses and VHVs

To meet the increased demand for services, VHVs conducted home visits to encourage individuals to undergo blood pressure screening; however, these efforts were often met with disinterest or disrespect. One VHV with personal experience managing hypertension noted that she encouraged individuals and their family members to seek early medical evaluation at the hospital, but her advice was frequently disregarded. Consequently, health care workers described delays in diagnosis and the adverse consequences of poorly controlled hypertension. Health care workers stated that some participants expressed a lack of trust in nurses and VHVs, choosing instead to disclose their symptoms only to physicians and to follow physicians’ advice. This mistrust hindered community nurses from effectively assessing and managing the health of individuals with hypertension.

### Theme 4: Enhancing Enablers and Overcoming Barriers to Change Behavior

Individuals with prehypertension identified several challenges they needed to overcome to take proactive action and improve their blood pressure control. Many perceived these to be insurmountable due to work schedules that limited their time for physical activity and healthy food preparation. In this context, family support was considered essential to enable at-risk individuals to adopt and maintain health-promoting behaviors.

#### Subtheme 4.1: Benefits of Complying with Blood Pressure Control

A few participants perceived benefits in adhering to recommended lifestyle changes that supported better blood pressure control. Consumers with hypertension reported perceived benefits from attending regular follow-up appointments, including physical examinations, receiving updated knowledge on blood pressure management, and adhering to prescribed medications. It also appeared that individuals with hypertension had more opportunities to access health information than those with prehypertension.

#### Subtheme 4.2: Tailored Health Education is Essential to Promote Behavior Change

Factors that prompt or trigger behavior change are labeled in the HBM as cues to action, which may be internal or external, for example, pain, headaches, health education from health care providers, or the experience of serious illness in family members. Health care workers noted that currently available health education materials promoting strategies to achieve better blood pressure control lacked variety and often did not reach those working in factory settings. Furthermore, some consumer participants reported difficulty remembering all the details from the health education they had received, and this may have contributed to their inability to apply the information effectively in their daily lives.

#### Subtheme 4.3: Individual Ability to Reduce Barriers to Exercise

Self-efficacy is an individual’s confidence in their ability to succeed in behavior change, such as maintaining regular exercise, regardless of perceived obstacles. Despite the reported barriers to exercise participation, some consumer participants demonstrated the ability to engage in physical activity and explore alternative forms of exercise, despite occasional limitations. For example, 1 participant with knee pain adapted by choosing arm-swinging exercises instead of walking or cycling.

## Discussion

Using the HBM as a framework, the perceptions of key local stakeholders and consumers regarding barriers and facilitators influencing the uptake of behavior change were explored in this study. Community strengths identified as key factors in improving good blood pressure control included the availability of government funding for primary health care services, and the availability of exercise equipment provided by the municipality. Barriers to successful behavior change included busy work schedules, concerns about personal safety and environmental conditions, and limited appreciation of the risks associated with having poor blood pressure control.

Similar to previous studies conducted in Southeast Asia, contextual and cultural factors were the most influential barriers inhibiting the maintenance of healthy lifestyle behaviors among individuals with prehypertension.^14^ Individuals diagnosed with hypertension exhibited higher levels of activation and participation in actions aimed at improving blood pressure control. In contrast, individuals with prehypertension showed lower levels of engagement in behavior change, indicating a limited awareness or appreciation of the importance and effectiveness of health prevention. In line with the HBM, individuals are more likely to engage in health-related action when they perceive susceptibility to a condition, believe the condition has serious consequences, perceive the benefits of action, and judge these benefits to outweigh perceived barriers.^21^

Individuals with prehypertension were more likely to take action to prevent hypertension if they perceived adopting healthy behaviors as less difficult.^14,20,23,26^ In this study, participants identified various barriers to adopting healthy behaviors, including work patterns, weather conditions, an unsafe environment, and distrust of health care providers. Most individuals with prehypertension in this community were general employees, particularly factory workers. These findings align with previous studies showing that busy daily routines leave individuals with little time to prepare healthy meals or engage in physical activity, which is perceived as an almost insurmountable barrier to adopting health-promoting behaviors.^34-37^ Similarly, workplace-based studies have reported that internal barriers to adopting healthy eating habits include lack of self-control, the convenience of unhealthy options, and limited access to healthy foods.^34,36,37^

Weather conditions in tropical regions such as high temperatures during the summer and heavy rainfall during the rainy season are associated with global warming and can affect individuals’ ability to engage in physical activity.^18,19^ According to Koontalay et al,^18^ Thailand’s tropical climate affects individuals with cardiovascular disease, particularly in terms of exercise and daily lifestyle behaviors. The impact of weather on exercise participation is similar to that observed by Ferguson et al,^38^ who found that in Western countries, extreme low temperatures, rainfall, and high wind speeds were negatively associated with engagement in moderate-to-vigorous physical activity. Recently, Thailand has experienced adverse weather conditions and significant air pollution, with high levels of Particulate Matter (PM) 2.5. These environmental issues hinder people from exercising outdoors, even in community-provided spaces. This is in line with findings from systematic reviews and cross-sectional studies that show exposure to air pollutants—regardless of whether the levels are low or high—increases the prevalence, hospitalization, disability, mortality, and associated costs of cardiovascular disease.^39,40^ Additionally, the coronavirus disease (COVID-19) pandemic has been a major factor restricting participation in group exercise activities.^41^ Furthermore, some participants reported feeling unsafe when using community gyms alone.

Social, cultural, and economic factors such as food costs and affordability in Southeast Asian countries are key barriers to achieving healthy diets for managing hypertension.^15-18^ In Northern Thailand, local cuisine frequently features high fat and sodium condiments such as fermented fish (*Pla ra*), fermented soybeans (*Thua nao*), and shrimp paste, which are regularly used in daily cooking and in dishes prepared for community events. Our findings align with previous studies showing that several Asian dishes are high in sodium due to the use of traditional fermented foods that are naturally high in salt, the addition of bouillon cubes and monosodium glutamate during food preparation, and the common practice of adding extra salt to already prepared meals.^42,43^ Additionally, some participants reported that they could not refuse or choose healthier food options when eating at community events, even if they believed the food was unhealthy. This is likely due to social norms in rural communities, where refusing to eat food may be perceived as a sign of disgust or disrespect toward the hosts and the community event.

Findings from health care workers indicate that some participants distrusted the information provided and showed low adherence to advice from nurses and VHVs. VHVs may be perceived as lacking credibility, while nurses often have limited interactions due to time constraints and the lack of private space. This aligns with an umbrella review showing that community health volunteers are effective in health promotion but less so in complex tasks requiring professional training.^44^ Continuous training on hypertension signs and communication skills is recommended to strengthen the capacity and confidence of those supporting people with prehypertension in the community.^44,45^

This study highlights that overcoming barriers can be achieved by increasing perceived benefits, supporting appropriate health promotion interventions, and empowering individuals to maintain or adopt healthy behaviors. This may be because individuals with prehypertension are more likely to comply with hypertension prevention information if they believe the benefits outweigh the challenges. For example, they may perceive that maintaining healthy behaviors can prevent hypertension and increase life expectancy. Maintaining healthy behaviors can be supported by strengthening self-efficacy. This finding is consistent with an integrative review showing that self-efficacy has small-to-large associations with physical activity and dietary behaviors.^46^ Moreover, cues to action from health professionals, family, and community members play a key role in motivating and supporting behavior change. These findings are consistent with previous studies on hypertension prevention, which suggest that nursing interventions based on the HBM should focus on addressing barriers and strengthening confidence and motivation for lifestyle modification.^14,23,26^ In addition, tailored nursing interventions for hypertension prevention should be designed to reflect the context, culture, and health literacy of each target group. Rural populations often have diverse backgrounds and circumstances. Prior studies have emphasized that interventions should be adapted to the specific needs, health literacy levels, and preferences of different communities, using varied delivery methods and media to improve effectiveness and accessibility.^14,16,47,48^

In our study, participants perceived that both susceptibility and severity of hypertension was increasing in their community. They noted that the number of individuals with prehypertension in the community is increasing and that this will have consequences for health care delivery, families, and individuals’ own health. Additionally, some individuals with prehypertension in our study were not aware of their condition and did not participate in health screening or adopt healthy behaviors. This may be because people at risk of hypertension often do not experience symptoms and are not required to take medication, which may lead them to disregard hypertension-related information and recommendations. Similarly, previous studies have shown that perceived susceptibility is crucial for ensuring compliance with health recommendations, as individuals can have a condition without experiencing any symptoms.^20,22^ Although our findings suggest that perceived threat may not be the most important factor, community nurses and health care providers should emphasize the consequences of high blood pressure and invite individuals with negative experiences of uncontrolled blood pressure to share their real-life stories and management strategies to increase self-awareness.

The findings may be transferable to other settings with similar socioeconomic and health care structures. This study illustrates how cultural, medical, and socioeconomic factors intersect to shape participants’ experiences of hypertension and prehypertension. Cultural dietary practices, including traditional foods high in sodium, and social norms common in Southeast Asia further constrain individuals’ ability to adopt healthy behaviors. Within this context, medical factors—such as the high burden of hypertension and prehypertension—exacerbate difficulties in achieving blood pressure control, highlighting the need for support from multidisciplinary teams and collaboration with local community sectors in health care delivery. Socioeconomic conditions in many LMICs also limit access to healthy foods and opportunities for regular physical activity.

### Strengths and Limitations

The key strength of this study is that it is the first to apply the HBM to assess the effectiveness of health promotion activities in rural communities in Thailand, helping to identify and highlight important gaps in health service delivery. First, this is an exploration of the diverse perspectives of local stakeholders, providing a comprehensive overview of the barriers and facilitators related to community management, the health care system, service delivery, and health behaviors among individuals with prehypertension. The main limitation of this study is that it was conducted in a single rural setting, which may limit the transferability of the findings to other areas. Second, the sample size of consumer participants was small (n = 7), which may have limited the depth of information on individuals with hypertension and prehypertension. However, given the high prevalence of hypertension in rural Thailand,^49^ this study also included 9 community leaders and 6 health care providers who contributed relevant hypertension-related perspectives. Some of these participants had hypertension themselves, while others had experience caring for people with hypertension. Together, these groups provided additional insight into the experiences of individuals with hypertension. Third, the HBM is an intrapersonal-level behavior theory and may be limited in its ability to explain behavior at the community and social levels.

### Implications for Nursing Practice

The HBM is a useful framework to explain individuals’ responses to health promotion messaging and its effectiveness in achieving behavior change. These findings suggest that community nurses should focus on exploring perceptions of severity, susceptibility, barriers, benefits, cues to action, and self-efficacy related to the adoption of healthy behaviors for preventing hypertension. Additionally, community nurses can use this information to codesign patient-centered and culturally tailored interventions that address specific needs and health literacy levels. To be effective when working in the community, nurses cannot work alone. This study highlights that community leaders and VHVs are key stakeholders in supporting resources and driving interventions forward. For example, community leaders can help develop safe environments for physical activity and advocate for public health policies—such as promoting healthy markets to support affordable access to nutritious food and encouraging the use of local herbs instead of high-sodium condiments. Future research should involve codesigning tailored interventions based on the real situations in each community, in collaboration with local key stakeholders.

## Conclusion

Local key stakeholders and consumers identified that local contextual and cultural factors rather than a lack of awareness were the main barriers to successful behavior change in their community. To overcome these barriers, there is a need for community level interventions, such as promotion of participatory healthy public policy, provision of safe places to exercise, and cooking classes that increase local consumers’ skills in preparation of lower salt but appetizing cuisine and person-level interventions that provide cues to action that promote integration of healthy lifestyle choices into individual’s daily routines.

## Supplemental Material

sj-docx-1-wjn-10.1177_01939459261432411 – Supplemental material for Using the Health Belief Model to Explore Behavior Change in the Community to Improve Blood Pressure Control: A Qualitative Study Supplemental material, sj-docx-1-wjn-10.1177_01939459261432411 for Using the Health Belief Model to Explore Behavior Change in the Community to Improve Blood Pressure Control: A Qualitative Study by Pataporn Bawornthip, Jo Mcdonall, Decha Tamdee, Andrea Driscoll and Anastasia Hutchinson in Western Journal of Nursing Research
