# Supplementary material for: Using the Health Belief Model to Explore Behavior Change in the Community to Improve Blood Pressure Control: A Qualitative Study
Source: West J Nurs Res. 2026 Apr 24;48(7):729–40. doi: 10.1177/01939459261432411 (PMC13254157; doi:10.1177/01939459261432411)
Supplement: sj-docx-1-wjn-10.1177_01939459261432411 – Supplemental material for Using the Health Belief Model to Explore Behavior Change in the Community to Improve Blood Pressure Control: A Qualitative Study [file sj-docx-1-wjn-10.1177_01939459261432411.docx]

**Supplementary Table S1: Semi-structured interview for focus group local stakeholders**

| **Local key stakeholders** | **Semi structured interview** |
| --- | --- |
| 1. Health service leaders at a municipal, village leader level | 1. What is the situation of hypertension/prehypertension in the community?  2. What is the policy related to the management of prehypertension in the community?  3. What is the budget support policy for the management of prehypertension in the community?  4. What are the activities/projects related to the prevention of hypertension in the community?  5. What are the barriers in implementing the prevention of hypertension in the community?  6. What are the strengths, needs, facilitators in implementing the prevention of hypertension in the community? |
| 2. Health care provider, village health volunteer | 1. What is the situation of hypertension/prehypertension in the community?  2. What is the number of patients with hypertension and prehypertension in the past 5 years (2019-2023)?  3. What are the implications of the increasing number of people with hypertension?  4. What is, or should there be a policy on the management of groups at high-risk of developing hypertension of sub-district health promoting hospitals?  5. What is or should there be a budget to support health promotion and management of people with prehypertension?  6. What is the level of organisational support for developing programs focused on modifying exercise behavior and food consumption for people with prehypertension? and where is the source of support from?  7. What are the implementation/activities/projects related to the prevention of hypertension in the community? or what activities should we have?  8. What are the barriers in implementing the prevention of hypertension in the community?  9. What are the strengths, needs, facilitators in implementing the prevention of hypertension in the community? |
| 3. People with hypertension, people with prehypertension, family members | 1. How have you been affected by having high blood pressure/prehypertension?  *(Effects of hypertension can be divided into 3 aspects: physical, mental, socio-economic such as discomfort from headache, anxiety, stress, work, income, social activities (e.g., temples, funerals, weddings, etc.).*  2. Diet behaviours  2.1 What foods do you like to eat, such as taste? What are your favorite foods that you eat often?  2.2 What are the condiments that you usually use in cooking?  2.3 Do you cook yourself or buy? Who cooks for you?  2.4 What encourages you to consume food to prevent hypertension?  2.5 What are the barriers that induced you not to consume healthier foods to prevent hypertension?  3. Exercise  3.1 What type of exercise do you enjoy, how often, and how long?  3.2 Do you exercise to the point of exhaustion by breathing hard and fast?  3.3 What encourages you to exercise?  3.4 Is there a place and equipment for exercise in your community? Do you need to pay for using the service or not?  3.5 What are the barriers that mean you do not exercise?  4. How have you been affected by having a family member suffering from hypertension or prehypertension? (Mental, economic, social)  5. Have you ever been educated about hypertension, health behaviours to prevent hypertension? what source?  6. Have you ever participated in activities/projects related to changing health behaviors to prevent hypertension? |

**Supplementary Figure S1: Code tree**

**CODE TREE**

Perceived benefits, cues to action and self-efficacy

Perceived severity

Perceived barriers

Perceived susceptibility

1.Lack of knowledge about the diagnosis of hypertension and risk groups

2. No sign and symptoms of high blood pressure

2.1 Community members do not perceive themselves to be at risk.

2.Not coming for health check-ups.

2.2 The importance of disease prevention is not widely understood, and people do not understand the consequences of NCDs.

1.Lack of awareness of the disease

1.2 The exponential growth in NCDs is made visible in our overcrowded clinics.

3.Share information to family members

1.Adherence to appointments and medication

4.Invite a person with direct experience to share their experience.

3.Emphasizing the involvement of family members.

2.Improve self-awareness

6.Access to low-sodium seasonings

5.Meal planning and cooking demonstrations

2.Setting goals for behavioral change

3. Municipal financial support

1.Facilities and equipment for exercise.

2.Receiving ongoing health education by healthcare professionals.

1.Provide health education using appropriate strategies and media

3.Performance of village health volunteers

2.Perception of authority

1.Patient trust in physicians

5.Habit of purchasing seasonings

4.Use of seasonings or condiments

3.Local dishes prepared with high-sodium seasonings

2.Inappropriate eating behaviours

1.An abundance of both healthy and unhealthy food options within the community

2.Unsafe gym

1.Air pollution and bad weather

3.1 Working patterns and busy lifestyle pose challenges to adopting healthy behaviours.

5.Self-adjustment of antihypertensive medication without medical consultation

4.Health consequences of hypertension

3.Factors contributing to high blood pressure

5.The strength or capacity of the community and staff.

4.Develop a monitoring system for individuals at risk of hypertension

3.Emotions or sentiments of village health volunteers.

2.Access to services for individuals at risk of hypertension

3.At-risk groups lack of time (obstacle to activities)

2.Ignoring one's health

8.Psychological impact on family members.

7.Effects of fluctuating blood pressure on engagement in community activities

6.Symptoms experienced by hypertensive patients during periods of blood pressure instability

1.Prompt and timely access to healthcare services

2.The incidence of hypertension and the number of people

1.The number of patients and at-risk groups is increasing.

1.1 Hypertension is hidden but its consequences impact on family life and community.

1.Work-related lifestyle changes

3.2 Adverse weather conditions, disease outbreaks and lack of environmental safety make it difficult for individuals to maintain an exercise program.

1. Community perceptions of the rising prevalence of hypertension and its associated health and social consequences

4.2 Tailored health education is essential to promote engagement in behaviour change.

4.3 Individual ability to reduce barriers to exercise.

4.1 Benefits of complying with blood pressure control

3.4 Lack of trust in information and low adherence to advice from nurses and village health volunteers.

3.3 Traditional foods, particularly those served and seasoned during festivals, are delicious but often high in sodium and fat.

4. Enhancing enablers and overcoming barriers to behaviour change.

3. Contextual and cultural barriers challenging the adoption of healthy lifestyles in everyday life.

2. High blood pressure is asymptomatic, as a consequence there is little motivation for behaviour change
